# Exogenous and Endogenous Triggers Differentially Stimulate *Pigr* Expression and Antibacterial Secretory Immunity in the Murine Respiratory Tract

**DOI:** 10.1007/s00408-021-00498-8

**Published:** 2021-11-26

**Authors:** Alexander Pausder, Jennifer Fricke, Klaus Schughart, Jens Schreiber, Till Strowig, Dunja Bruder, Julia D. Boehme

**Affiliations:** 1 Research Group Infection Immunology, Institute of Medical Microbiology and Hospital Hygiene, Health Campus Immunology, Infectiology and Inflammation, Otto-Von-Guericke-University, Leipziger Strasse 44, 39120 Magdeburg, Germany; 2 Research Group Immune Regulation, Helmholtz Centre for Infection Research, Braunschweig, Germany; 3 ESF Graduate School ABINEP, Magdeburg, Germany; 4 Research Group Infection Genetics, Helmholtz Centre for Infection Research, Braunschweig, Germany; 5 University of Veterinary Medicine, Hannover, Germany; 6 University of Tennessee Health Science Center, Memphis, TN USA; 7 Experimental Pneumology, Health Campus Immunology, Infectiology and Inflammation, University Hospital for Pneumology, Otto-Von-Guericke-University, Magdeburg, Germany; 8 Department of Microbial Immune Regulation, Helmholtz Centre for Infection Research, Braunschweig, Germany; 9 Current Address: Research Group Nanoinfection Biology, Helmholtz Centre for Infection Research, Braunschweig, Germany

**Keywords:** Secretory immunity, Respiratory tract, Polymeric immunoglobulin receptor, Immune modulation, Infection

## Abstract

**Purpose:**

Transport of secretory immunoglobulin A (SIgA) through the airway epithelial cell barrier into the mucosal lumen by the polymeric immunoglobulin receptor (pIgR) is an important mechanism of respiratory mucosal host defense. Identification of immunomodulating substances that regulate secretory immunity might have therapeutic implications with regard to an improved immune exclusion.

Thus, we sought to analyze secretory immunity under homeostatic and immunomodulating conditions in different compartments of the murine upper and lower respiratory tract (URT&LRT).

**Methods:**

*Pigr* gene expression in lung, trachea, and nasal-associated lymphoid tissue (NALT) of germ-free mice, specific pathogen-free mice, mice with an undefined microbiome, as well as LPS- and IFN-γ-treated mice was determined by quantitative real-time PCR. IgA levels in bronchoalveolar lavage (BAL), nasal lavage (NAL), and serum were determined by ELISA. LPS- and IFN-γ-treated mice were colonized with *Streptococcus pneumoniae* and bacterial CFUs were determined in URT and LRT.

**Results:**

Respiratory *Pigr* expression and IgA levels were dependent on the degree of exposure to environmental microbial stimuli. While immunostimulation with LPS and IFN-γ differentially impacts respiratory *Pigr* expression and IgA in URT *vs*. LRT, only prophylactic IFN-γ treatment reduces nasal colonization with *S. pneumoniae*.

**Conclusion:**

Airway-associated secretory immunity can be partly modulated by exposure to microbial ligands and proinflammatory stimuli. Prophylactic IFN-γ-treatment modestly improves antibacterial immunity in the URT, but this does not appear to be mediated by SIgA or pIgR.

**Supplementary Information:**

The online version contains supplementary material available at 10.1007/s00408-021-00498-8.

## Introduction

Airway epithelial cells (AECs) constitute the first line of defense against respiratory pathogens. They express transmembrane proteins, which form tight junctions that allow only small ions or water to traverse paracellularly [1]. Claudin and occludin are vital for epithelial defense [2] and altered claudin expression affects airway epithelial barrier function [3, 4]. Furthermore, AECs constitutively secrete antimicrobial proteins, complement factors and cytokines, and rapidly mount antimicrobial immune responses upon inflammatory and infectious stimuli [5–9]. Importantly, AECs have a central function in antibody-mediated mucosal immunity. Multimeric IgA and IgM are actively transported through AECs via the polymeric immunoglobulin receptor (PIGR) and are secreted into the mucosal lumen as secretory immunoglobulins (SIgs) [10]. Especially SIgA is known to prevent pathogen adhesion, thus averting microbial infiltration [11]. Moreover, SIgA plays a crucial role in the regulation of *Streptococcus pneumoniae* nasal colonization in mice [12].

In this context, *Pigr* deficiency manifests in susceptibility to mycobacterial respiratory infections [13] and development of a COPD-like phenotype driven by an altered lung microbiome and bacterial invasion of the airway epithelium [14]. The importance of SIgA for airway homeostasis is furthermore highlighted by the findings of SIgA deficiency in small airways of COPD patients, which is associated with persistent inflammation and airway wall remodeling [15]. Moreover, chronic airway diseases reduce PIGR expression in the bronchial epithelium resulting in increased disease severity (COPD) and impaired SIgA-mediated mucosal defense (asthma) [16, 17]. While the key role of PIGR and secretory immunity for airway homeostasis is undisputable, knowledge on their expression and regulation in the airways is still fragmentary. Since targeted modulation of secretory immunity represents an interesting option to improve immune exclusion of respiratory pathogens, we here aimed to further dissect PIGR-mediated immunity in the airways with the specific focus on the applicability of exogenous and endogenous stimuli to regulate this aspect of humoral antimicrobial defense.

## Methods

### Mice

BALB/c and C57BL/6 J mice (age: 11–46 weeks) were maintained in individually ventilated cages (IVCs) under specific pathogen-free (SPF) conditions at the Helmholtz Centre for Infection Research (HZI), Braunschweig. Germ-free mice (C57BL/6 N, age: 10 weeks) were bred and maintained in isolators in a germ-free (GF) facility (HZI). C57BL/6 J mice with an undefined microbiome (maintained in open cages, age: 10–18 weeks) were provided by Dirk Schlüter (Otto-von-Guericke-University [OvGU], Magdeburg). For pneumococcal colonization experiments female C57BL/6JRj mice (age: 12 weeks) were purchased from Janvier Labs (France) and maintained in IVCs under SPF conditions (OvGU).

### Treatment with Immunomodulating Substances

BALB/c, C57BL/6 J, and C57BL/6JRj mice were treated intranasally (i.n.) with LPS (Sigma-Aldrich, Germany), (10 µg/ 25 µl PBS or solvent alone) or recombinant murine IFN-γ (Peprotech, Germany), (1 µg/ 20 µl ddH_2_O with 5% BSA or solvent alone). BALB/c and C57BL/6 J mice were sacrificed 1 or 2 days post-treatment. Lung, trachea, NALT, BAL, NAL, and serum were collected. Organs were used for RNA isolation and qPCR. Fluids were used for ELISA analysis. Blood was collected by cardiac puncture. BAL fluid was collected by flushing the lungs with 1 ml PBS via the trachea. The nasopharynx was flushed with 1 ml PBS via the trachea and NAL fluid was collected at the nostrils.

### Pneumococcal Infection

*Streptococcus pneumoniae* serotype 19F (strain BHN100) [18] was provided by Birgitta Henriques-Normark (Karolinska Institutet, Stockholm). Bacteria were grown in Todd-Hewitt yeast (THY) medium as previously described [19]. LPS- and IFN-γ-treated C57BL/6JRj mice and control groups were infected i.n. with 10^8^ *S. pneumoniae* 19F in 10 µl PBS 48 h after the first treatment. Mice were sacrificed 18 h post-infection and lung, trachea, NALT, and nasopharynx were homogenized using a tissue homogenizer (KINEMATICA AG, Switzerland). Samples were plated onto Columbia blood agar plates (BD Diagnostic Systems, Germany) and incubated over night at 37 °C, 5% CO_2_. CFU were counted to determine the bacterial burden.

### Quantitative Real-Time PCR (qPCR)

RNA was isolated from lung, trachea, NALT, and MLE-15 cells using RNeasy Plus Mini Kit (QIAGEN, Germany). cDNA was synthesized from 1 µg of RNA using Oligo dT Primers (Thermo Fisher Scientific, USA), Random Primers (Thermo Fisher Scientific, USA), dNTP-Mix (10 mM), and SuperScript™ III Reverse Transcriptase (Thermo Fisher Scientific, USA). QPCR was performed using the SensiFAST™ SYBR® No-ROX Kit (Bioline, USA). Temperature profile: 95 °C for 2 min, 40 cycles at 95 °C for 5 s, 60 °C for 10 s, and 72 °C for 5 s. ß-Actin (*Actb*) served as reference gene. Primer sequences: *Pigr* forward: 5ʹ-GTGCCCGAAACTGGATCACC-3ʹ, *Pigr* reverse: 5ʹ-TGGAGACCCCTGAAAAGACAGT-3ʹ, *Actb* forward: 5ʹ-ACACCCGCCACCAGTTCG-3ʹ, *Actb* reverse: 5ʹ-GTCACCCACATAGGAGTCCTTC-3ʹ, *Cldn-7* forward: 5ʹ-AGCGAAGAAGGCCCGAATAG-3ʹ, *Cldn-7* reverse: 5ʹ-AGGTCCAAACTCGTACTTAACG-3ʹ, *Cldn-18* forward: 5ʹ-GACACCAGATGACAGCAACTTC-3ʹ, *Cldn-18* reverse: 5ʹ-TTCATCGTCTTCTGTGCGGG-3ʹ, *IgJ* forward: 5ʹ-GCATGTGTACCCGAGTTACC-3ʹ, *IgJ* reverse: 5ʹ-TTCAAAGGGACAACAATTCGG-3ʹ, and *CD19* forward: 5ʹ-CCTGGGCATCTTGCTAGTGA-3ʹ, *CD19* reverse: 5ʹ-CGGAACATCTCCCCACTATCC-3ʹ. Expression of target genes in relation to reference gene was determined using the 2^−∆∆CT^ method.

### Enzyme-Linked Immunosorbent Assay (ELISA)

Relative IgA levels (including monomeric IgA, dimeric IgA, and SIgA molecules) in BAL, NAL, and serum were determined by ELISA using a monoclonal rat anti-mouse IgA capture antibody (Southern Biotech, USA) in combination with a polyclonal rabbit anti-mouse IgA secondary antibody (Abcam, UK) and a polyclonal swine anti-rabbit, HRPO-linked detection antibody (Dako, UK).

### In Vitro Stimulation

MLE-15 cells were cultivated in Dulbecco’s Modified Eagle’s Medium (DMEM, Gibco, USA) supplemented with 4.5 g/l glucose, 10% FBS (Biowest, USA), and 1% penicillin/streptomycin (Gibco, USA). 3 × 10^5^ cells were seeded in 12-well plates and incubated in 1 ml medium over night at 37 °C, 5% CO_2_. Cells were washed using 1 ml DMEM (4.5 g/l glucose, no additives). Recombinant murine IFN-γ (Peprotech, Germany) was diluted in DMEM (w/o additives) and added to the cells. After 24 h supernatants were removed and RNA was isolated from the cells and used for qPCR.

### Statistical Analysis

Statistical analyses were performed either by two-tailed, unpaired t test (Gaussian distribution, two groups), Mann–Whitney test (no Gaussian distribution, two groups), one-way ANOVA (Gaussian distribution, more than two groups, post-test: Bonferroni’s Multiple Comparison Test), or Kruskal–Wallis test (no Gaussian distribution, more than two groups, post-test: Dunn’s Multiple Comparison Test) using GraphPad Prism software (GraphPad Software Inc., USA, Version 5.04).

## Results

**Fig. 1:**
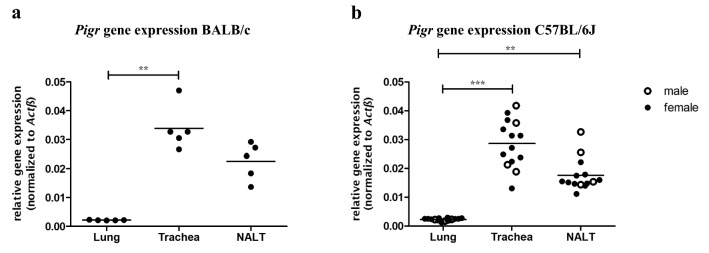
Basal *Pigr* expression in the airways. RNA from lung tissue, trachea, and nasal-associated lymphoid tissue (NALT) of **a** BALB/c mice (*n* = 5) and **b** C57BL/6 J mice (*n* = 14) was isolated and reversely transcribed into cDNA. *Pigr* expression was assessed by qPCR. *Actb* served as reference gene (data for individual mice are graphed; mean is indicated by horizontal line; ** for *p* ≤ 0.01; *** for *p* ≤ 0.001)

In order to determine differences in *Pigr* gene expression in the upper (URT) and lower respiratory tract (LRT) and its dependency on genetic background, sex, and age, we initially compared *Pigr* expression patterns in commonly used mouse strains and different sexes. BALB/c (Fig. 1a) as well as C57BL/6 J mice (Fig. 1b) exhibit highest *Pigr* expression in the trachea, followed by nasal-associated lymphoid tissue (NALT) and lung. While pulmonary *Pigr* expression levels were relatively constant, higher variations were detected in trachea and NALT. Moreover, comparative analyses of *Pigr* expression in BALB/c *vs.* C57BL/6 J mice (Fig. S1a) and male vs. female C57BL/6 J mice (Fig. S1b) revealed no significant differences. We also analyzed whether *Pigr* gene expression is age-dependent (Fig. S2a–c) but found no such correlations. Together, these data demonstrate marked tissue-specific differences in *Pigr* gene expression, which were however independent of genetic background, sex, and age.

**Fig. 2:**
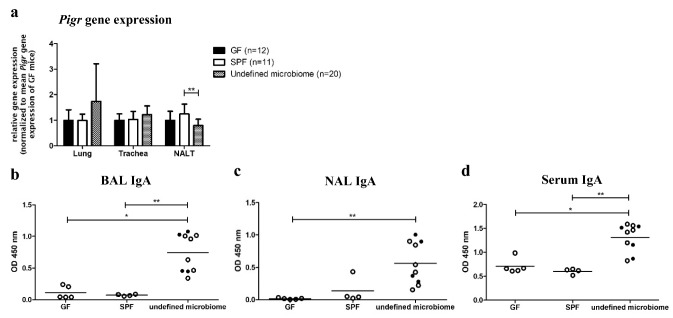
*Pigr* expression and secretory immunity in differentially colonized mice. RNA from lung tissue, trachea, and NALT of specific pathogen-free (SPF), germ-free (GF), and mice with an undefined microbiome was isolated and reversely transcribed into cDNA and qPCR analysis was performed (● indicates female, ○ indicates male). **a** *Pigr* expression normalized to *Actb*. Normalized gene expression values of each organ were divided by the mean gene expression of the GF mice for the respective organ (mean expression values ± SD are graphed). IgA levels were determined in **b** bronchoalveolar lavage (BAL), **c** nasal lavage (NAL), and **d** serum of GF (*n* = 5), SPF (*n* = 4), and mice with an undefined microbiome (*n* = 10) by semi-quantitative ELISA (data for individual mice are graphed; mean is indicated by horizontal line; * for *p* ≤ 0.05; ** for *p* ≤ 0.01)

To determine whether *Pigr* expression and secretory immunity in the airways were influenced by microbial exposure we compared germ-free (GF) mice (no microbial exposure), SPF mice (IVCs, exposure to a limited microbial flora), and mice with an undefined microbiome (open cage maintenance, highest degree of exposure to airborne microorganisms). While similar *Pigr* expression levels were observed in the LRT of all three experimental groups, we detected significantly lower *Pigr* expression in the NALT of mice with an undefined microbiome compared to SPF mice (Fig. 2a). In contrast to the unaltered (lung, trachea) or even reduced (NALT) *Pigr* expression levels in mice with an undefined microbiome, we detected significantly increased IgA concentration in the LRT (Fig. 2b) and URT (Fig. 2c) in this group, which was associated with a systemic IgA increase (Fig. 2d).

**Fig. 3:**
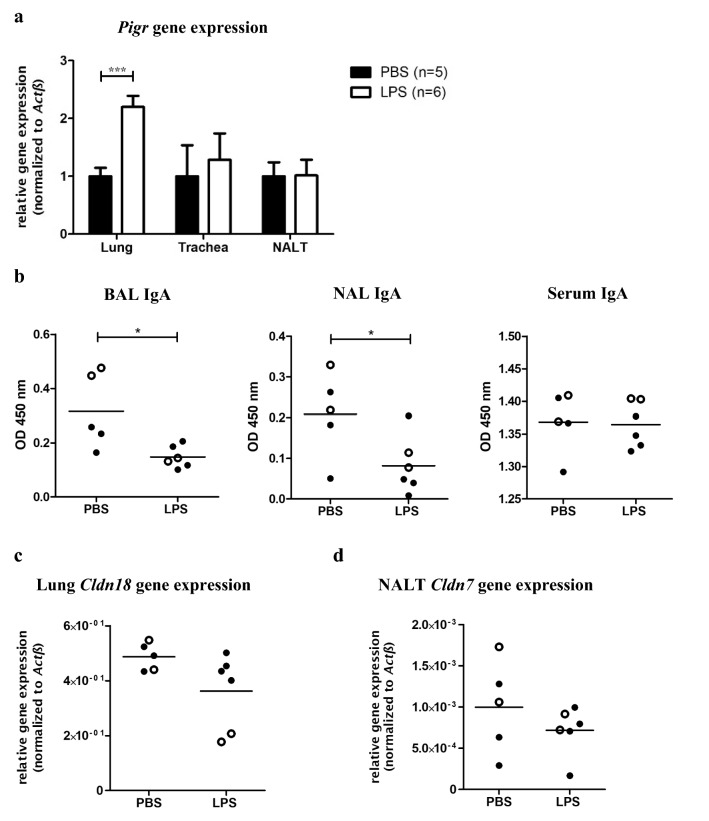
Effect of LPS treatment on airway secretory immunity and epithelial barrier function. BALB/c mice were treated i.n. with 10 µg of LPS (*n* = 6) or solvent alone (*n* = 5). 48 h post-treatment RNA from lung tissue, trachea, and NALT was isolated and reversely transcribed into cDNA and qPCR analysis was performed (● indicates female, ○ indicates male). **a** *Pigr* expression normalized to *Actb*. Normalized *Pigr* expression values of each organ were divided by the mean *Pigr* expression of the PBS-treated mice for the respective organ (mean expression values ± SD are graphed). **b** IgA levels were determined in BAL, NAL, and serum by semi-quantitative ELISA. **c** *Cldn18* expression normalized to *Actb* and **d** *Cldn7* expression normalized to *Actb* (cumulative data from two experiments; data for individual mice are graphed; mean is indicated by horizontal line; * for *p* ≤ 0.05; *** for *p* ≤ 0.001)

Since in the intestine IgA production and *Pigr* expression are induced by microbial components [20–22], we investigated whether intranasal (i.n.) treatment of mice with LPS would affect airway *Pigr* expression and IgA levels. While *Pigr* expression in nose and trachea was not affected, we detected significantly increased expression in lung tissue 48 h after LPS treatment (Fig. 3a). Interestingly and in discordance with the increased pulmonary *Pigr* expression IgA levels in BAL and NAL fluid significantly decreased after LPS treatment. The amount of IgA in serum was however not affected (Fig. 3b). We thus speculated that LPS altered the barrier function of AECs, resulting in a decreased epithelial leakage. Therefore, we analyzed *Cldn* gene expression in lung and NALT. However, no significant differences in lung or NALT became apparent upon LPS treatment (Fig. 3c, d).

**Fig. 4:**
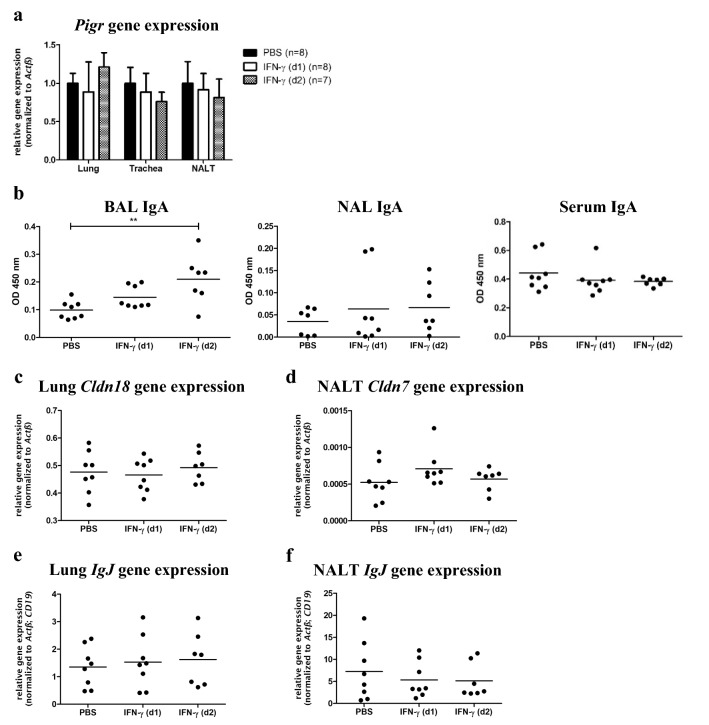
Effect of IFN-γ treatment on secretory immunity, epithelial barrier function, and B cells. BALB/c and C57BL/6 J mice were treated i.n. with 1 µg of IFN-γ (*n* = 8 for d1, *n* = 7 for d2) or solvent alone (*n* = 8). Lung, trachea, and NALT were removed on d1 or d2 post-treatment, RNA was isolated and reversely transcribed into cDNA, and qPCR analysis was performed. **a** *Pigr* expression normalized to *Actb*. Normalized *Pigr* expression values of each organ were divided by the mean *Pigr* expression of the PBS-treated group for the respective organ (mean expression values ± SD are graphed). **b** IgA levels were determined in BAL, NAL, and serum by semi-quantitative ELISA. **c** *Cldn18* expression normalized to *Actb*, **d** *Cldn7* expression normalized to *Actb*, and **e** + **f** *IgJ* gene expression normalized to *Actb* and *CD19* (cumulative data from two experiments; data for individual mice are graphed; mean is indicated by horizontal line; ** for *p* ≤ 0.01)

Next to LPS, Interferon-γ (IFN-γ) was shown to regulate human *PIGR* gene expression [23] and we confirmed the *Pigr*-inducing potential of this cytokine in murine AECs (Fig. S3). To assess possible effects of IFN-γ on PIGR-mediated secretory immunity in vivo, we analyzed airway secretory immunity in IFN-γ-treated mice. While IFN-γ did not affect *Pigr* expression after 48 h (Fig. 4a), the amount of pulmonary IgA increased after IFN-γ treatment. IgA levels in NAL and serum were however unaffected (Fig. 4b). We tested whether epithelial leakage might underlie the increased IgA levels by measuring *Cldn* gene expression in lung and NALT. *Cldn* gene expression was however not affected by single IFN-γ treatment (Fig. 4c, d). To investigate whether increased pulmonary IgA might arise from Ig-producing B cells which were induced by IFN-γ, we determined gene expression of the joining chain (*IgJ*) of multimeric IgA and IgM in lung and NALT. Nevertheless, airway *IgJ* expression was unaltered upon cytokine treatment (Fig. 4e, f), indicating that increased airway IgA levels are most likely not due to an IFN-γ-mediated increase of Ig-producing cells.

**Fig. 5:**
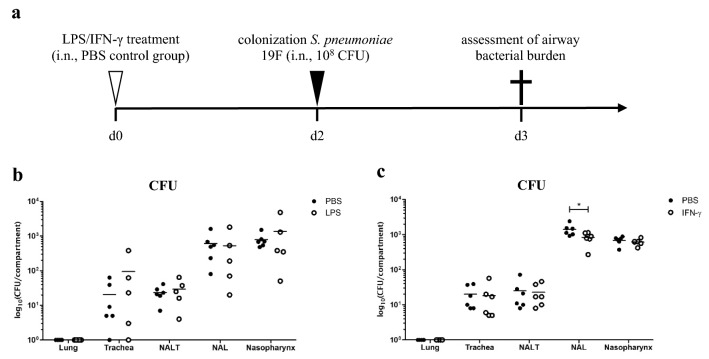
Effect of LPS or IFN-γ treatment on pneumococcal colonization in vivo. **a** Schematic representation of the experimental setup. **b** C57BL/6JRj mice were treated i.n. with 10 µg of LPS (*n* = 5) and control mice received PBS only (*n* = 6). **c** C57BL/6JRj mice were treated i.n. with 1 µg of IFN-γ (*n* = 6) and control mice received PBS only (*n* = 6). 2 days post-treatment all mice were i.n. infected with 10^8^ CFU of *S. pneumoniae* 19F and airway bacterial burden was assessed 18 h post-infection (individual data from one experiment are graphed; mean is indicated by horizontal line; * for *p* ≤ 0.05)

We finally tested whether modulation of airway secretory immunity by LPS- and IFN-γ treatment would ultimately affect antimicrobial defense. To this end, mice were i.n. treated with a single dose of LPS or IFN-γ. Two days post-treatment mice were inoculated with a colonizing strain of *S. pneumoniae* serotype 19F and airway bacterial burden was assessed (Fig. 5a). As expected, no pneumococci were detected in the lung tissue. While LPS treatment did not affect pneumococcal colonization (Fig. 5b), IFN-γ treatment led to significantly decreased nasal bacterial burden (Fig. 5c). These results demonstrate that at least in the URT mucosal immunity can be augmented by prophylactic IFN-γ treatment.

## Discussion

Previous studies on airway *Pigr* gene and PIGR protein expression mainly employed in vitro approaches [24, 25] or utilized tissue from patients with chronic respiratory diseases [16, 26–28]. Murine studies were either analyzing respiratory *Pigr* expression in the context of interleukin treatment [29, 30] or exposure to pathogen-associated molecules (Cholera toxin, amoeba lysates) [31]. These studies revealed that PIGR/*Pigr* expression is considerably influenced by exogenous and endogenous stimuli present in the airway microenvironment.

To our knowledge, we are first to report compartment-specific and sex-independent differences in basal airway *Pigr* gene expression levels in vivo. We observed that BALB/c as well as C57BL/6 J mice showed highest *Pigr* expression in trachea, followed by NALT and lung. We speculated that this originated from inherent differences in microbial density. Due to their anatomical localization, trachea and nasal cavities are more frequently exposed to microbial stimuli compared to the lung [32]. It is possible that a higher abundance of microbial ligands in the murine URT provide more signals triggering *Pigr* gene expression compared to the LRT, as well. This in turn might prevent bacterial spread from URT to LRT contributing to the relatively low bacterial density in the lung. Contrary to our expectations, we could not detect any alterations regarding *Pigr* expression between germ-free and microbially colonized mice, which suggests that *Pigr* expression in the murine respiratory tract is in fact unaffected by microbial colonization. However, as we assessed whole-tissue *Pigr* gene expression, we cannot fully exclude the possibility that signal dilution effects (*e.g.,* from leukocytes) affect compartment-specific *Pigr* expression in our analyses.

Commensal intestinal bacteria induce the production of IgA in mice [20]. Furthermore, it is known that lymphocyte numbers in nasal mucosa are dependent on housing conditions and exposure to microbial stimuli [33]. While those findings clearly highlight the impact of the microbiota on lymphocyte-associated mucosal immunity, the relationship between airway-associated secretory immunity and the level of microbial exposure is largely unknown. Our experiments revealed that *Pigr* expression was lower in NALT of mice with an undefined microbiome (highest microbial exposure) compared to mice maintained under SPF conditions. At the same time, airway and systemic IgA levels were increased in these mice. As fecal IgA levels depend on the composition of the intestinal microflora [34], it is conceivable that a similar effect might be present in murine airways. However, the fact that *Pigr* expression in NALT is reduced while IgA levels are increased indicates that there is likely no correlation between *Pigr* expression and IgA abundance at the whole-tissue level. However, we did not analyze *Pigr* expression exclusively in stromal cells but in whole tissue. Since it was shown that the microbial environment shapes cell composition in the mucosa, it is possible that an accumulation of leukocytes in mice with a high microbial exposure reduces the overall *Pigr* signal.

Previous studies revealed that human and murine intestinal epithelial cells exhibit increased *Pigr*/*PIGR* gene expression after LPS stimulation in vitro [22, 35, 36]. In line with this, we detected significantly increased pulmonary *Pigr* expression after LPS treatment. However, we also found that LPS did not alter overall *Pigr* expression in trachea or NALT. This might arise from the fact that bacterial colonization—and therefore exposure to *e.g.,* LPS—is more pronounced in the URT [32], resulting in a lower sensitivity of URT airway stromal cells to LPS. In contrast to this result, airway IgA levels were decreased in LPS-treated mice, while systemic IgA levels were unaffected. We hypothesized that decreased IgA levels resulted from decreased epithelial leakage and tested this by determining *Cldn18* and *Cldn7* expression. Claudins are major proteins that maintain epithelial barrier function and altered claudin expression results in altered AEC barrier function in the LRT (*Cldn18*) and URT (*Cldn7*) [2–4]. However, *Cldn18* and *Cldn7* expression were not affected by LPS, which disconfirmed our hypothesis. Since IgA binds LPS [37], it is possible that the administered LPS was already bound to IgA in the mucosal lumen. This might reduce the amount of detectable IgA, as the ELISA detects free IgA molecules with the highest functionality. However, the fact that *Pigr* expression in lung was increased while IgA in BAL was decreased and *Pigr* expression in NALT was unaltered while IgA in NAL was increased highlights that there is no consistent correlation between the two molecules in whole-tissue analysis of LPS-treated mice.

As LPS, IFN-γ induces *PIGR* expression in human epithelial cells [23, 38, 39]; however, its effect on *Pigr* and secretory immunity in vivo have not been addressed before. Despite no effect on airway *Pigr* expression, IFN-γ treatment increased pulmonary IgA levels. As *Cldn* expression was unaltered, we hypothesized that increased IgA concentrations after IFN-γ treatment might arise from mucosal B cells. Yet, airway *IgJ* expression was not affected, which suggests that activated B cells are most likely not the cause of increased airway IgA levels following IFN-γ stimulation. As mentioned before, we analyzed *Pigr* gene expression in tissues and not exclusively in stromal cells. It is known that intradermal IFN-γ injection stimulates intradermal lymphocyte migration in rats [40]. Thus, it is conceivable that i.n. IFN-γ treatment leads to the accumulation (and activation) of lymphocytes in the airways as well, which might reduce net *Pigr* expression. This might explain why there is no consistent correlation between *Pigr* expression and IgA levels in whole-tissue analysis of IFN-γ-treated mice.

As IgA is crucial for antimicrobial defense [15, 41–43], we investigated whether altered IgA levels upon LPS- and IFN-γ treatment correlated with altered antimicrobial immunity. We have chosen *S. pneumoniae* for experimental colonization of mice as it is one of the most relevant respiratory pathogens [44] and IgA is vital for antagonizing pneumococcal colonization and infection in vivo and in vitro [12, 45–47]. Indeed, prophylactic IFN-γ treatment significantly reduced nasal pneumococcal counts indicating improved antibacterial immunity. Since IFN-γ treatment led to increased IgA levels only in BAL and not in NAL, the effect IFN-γ has on colonization is most likely not mediated by its effect on IgA. Since IFN-γ triggers antibacterial activity in pulmonary macrophages [48] and macrophages are present in the murine NALT [49], it is conceivable that IFN-γ induces antibacterial activity in these cells as well. IFN-γ triggers the production of antibacterial molecules (e.g., β-defensins) [50]. Future studies will clarify whether IFN-γ-stimulated production of these antibacterial factors underlies the improved mucosal immunity in the URT.

In conclusion, our study demonstrates that secretory immunity in URT and LRT is differentially regulated by endogenous as well as exogenous stimuli. Further studies are needed to elucidate the underlying molecular frameworks as well as possible avenues for, e.g., prophylactic enhancement of airway mucosal immunity in infection-prone individuals.

## Supplementary Information

Below is the link to the electronic supplementary material.

Influence of genetic background and sex on Pigr expression. (a) BALB/c (n=5) vs. C57BL/6J mice (n=14) and (b) male (n=4) vs. female (n=10) C57BL/6J mice. RNA from lung tissue, trachea, and NALT of BALB/c and C57BL/6J mice was isolated and reversely transcribed into cDNA. Pigr expression was assessed by qPCR. Actb served as reference gene (data for individual mice are graphed; mean is indicated by horizontal line; n.s.: not significant) (eps 8494 KB)

Influence of age on Pigr expression. RNA from (a) lung tissue, (b) trachea, and (c) NALT of BALB/c mice (n=5) and C57BL/6J mice (n=14) was isolated and reversely transcribed into cDNA. Pigr expression was assessed by qPCR. Actb served as reference gene (data for individual mice are graphed; correlation analysis was performed by linear regression) (eps 7840 KB)

Pigr expression in IFN-γ-treated MLE-15 cells. Murine lung epithelial (MLE-15) cells (n=8 wells/experimental group) were treated with 1, 10, 100, or 1000ng of IFN-γ in 1ml medium or medium alone for 24h. RNA was isolated and reversely transcribed into cDNA. Pigr expression was assessed by qPCR analysis. Actb was used as reference gene (cumulative data from two experiments; data for individual cell culture wells are graphed and mean is indicated by horizontal line; *** for p ≤ 0.001) (eps 4993 KB)

## Data Availability

All data of this manuscript can be made available by the corresponding author.
